# Wnt ligand presentation and reception: from the stem cell niche to tissue engineering

**DOI:** 10.1098/rsob.170140

**Published:** 2017-08-16

**Authors:** Kate M. Mills, James L. A. Szczerkowski, Shukry J. Habib

**Affiliations:** Centre for Stem Cells and Regenerative Medicine, King's College London, London SE1 9RT, UK

**Keywords:** Wnt signalling, stem cell niche, tissue engineering, protein immobilization, cell therapy and regenerative medicine, adult and embryonic stem cells

## Abstract

Stem cells reside in niches where spatially restricted signals maintain a delicate balance between stem cell self-renewal and differentiation. Wnt family proteins are particularly suited for this role as they are modified by lipids, which constrain and spatially regulate their signalling range. In recent years, Wnt/β-catenin signalling has been shown to be essential for the self-renewal of a variety of mammalian stem cells. In this review, we discuss Wnt-responsive stem cells in their niche, and mechanisms by which Wnt ligands are presented to responsive cells. We also highlight recent progress in molecular visualization that has allowed for the monitoring of Wnt signalling within the stem cell compartment and new approaches to recapitulate this niche signalling *in vitro*. Indeed, new technologies that present Wnt in a localized manner and mimic the three-dimensional microenvironment of stem cells will advance our understanding of Wnt signalling in the stem cell niche. These advances will expand current horizons to exploit Wnt ligands in the rapidly evolving fields of tissue engineering and regenerative medicine.

## 1. Introduction

Stem cells are one of the fundamental underpinnings of tissue biology. They have the ability to self-renew and give rise to differentiated cells, replenishing a myriad of tissues with new cells throughout life. Stem cells are located in specialized microenvironments (also called niches) that impart the biochemical and biophysical cues required to support stem cell self-renewal and function. A spatially defined cellular niche often controls the number of stem cells. It also facilitates cellular differentiation as cells migrate away from the niche signals that promote self-renewal [1,2]. Wnt ligands (Wnts) have been identified as key signalling proteins in numerous embryonic [3,4] and adult stem cell niches including the intestine, liver, skin, brain, prostate and mammary gland, as outlined in table 1 [6,9,12,13,15,16,18,26,27]. Furthermore, Wnt ligands are often presented to recipient cells in a spatially restricted manner [12,27–30], a hallmark of niche signalling molecules, which typically act locally within a one- or two-cell diameter [1]. This is not surprising, as most studied Wnts are lipidated in the endoplasmic reticulum (ER), a modification that confers hydrophobicity [31] and restricts the diffusion range.

**Table 1.** Examples of Wnt-responsive stem/progenitor cells identified in murine tissues.

stem/progenitor cell	tissue	selected Wnt reporter mouse model	references
sub-ventricular zone neural stem cells	brain	Axin2-CreERT2/Rosa26mTmG Axin2-LacZ	[5] [6]
ciliary marginal zone (retina) cells	eye	TCF/Lef-LacZ/GFP-IRES-CreERT2 TCF/Lef-LacZ	[7] [8]
basal cells	interfollicular epidermis (skin)	Axin2-CreERT2/Rosa26mTmG	[9]
outer bulge cells	hair follicle	Axin2-CreERT2/Rosa26mTmG Lgr5-GFP-IRES-CreERT2/Rosa26-LacZ	[10] [11]
intestinal crypt base cells	intestine	Wnt3-HA Lgr5-LacZ Lgr5-GFP-IRES-CreERT2/Rosa26-LacZ	[12] [13]
basal pyloric cells	stomach	Lgr5-GFP-IRES-CreERT2/Rosa26-LacZ	[14]
renal precursor cells	kidney	Axin2-CreERT2/Rosa26mTmG	[15]
pericentral hepatocyte cells	liver	Axin2-CreERT2/Rosa26mTmG Lgr4-CreERT2/Rosa26-LacZ	[16] [17]
basal cells	mammary gland	Axin2-CreERT2/Rosa26mTmG Axin2-CreERT2/Rosa26-LacZ Procr-CreERT2-IRES-tdTomato/Rosa26mTmG	[18] [19]
taste progenitor cells	tongue	Lgr5-GFP-IRES-CreERT2/Rosa26-LacZ Lgr5-GFP-IRES-CreERT2/Rosa26-tdTomato	[20]
tympanic border cells (cochlear)	ear	Axin2-LacZ Axin2-CreERT2/Rosa26mTmG Lgr5-GFP-IRES-CreERT2/Rosa26-tdTomato	[21] [22]
luminal epithelial cells	prostate	Axin2-CreERT2/Rosa26mTmG	[23]
spermatogonial stem cells	testis	Axin2-CreERT2/Rosa26mTmG	[24]
osteoblast cells	bone	Axin2-CreERT2/Rosa26-ZsGreen	[25]

Wnt proteins are approximately 350 amino acids long and comprise a family of secreted signalling molecules. They have several shared features that are essential for activity, including multiple cysteine residues, a conserved serine residue for acetylation and lipidation, and a peptide sequence for secretion [27,32]. To date, 19 Wnt ligands have been identified in mammalian cells [33]. Wnt ligands contribute to pluripotency and stem cell self-renewal through activation of downstream signalling cascades including the Wnt/β-catenin pathway [34]. Stimulation of the Frizzled/LRP5/6 receptor complexes at the plasma membrane by a Wnt ligand activates Wnt signalling and causes the release of β-catenin from the destruction complex [35–37]. β-Catenin then translocates to the nucleus and binds to transcription factors TCF/LEF to stimulate transcription of Wnt target genes [38,39].

Wnt ligands are produced and secreted by a defined subset of cells within the niche. How these signals are presented to recipient stem cells is often dependent on tissue type and the Wnt ligands produced. Initially, we review the identification of mammalian Wnt-responsive stem/progenitor cells and use the intestine, liver and epidermis as examples. We discuss mechanisms of presenting Wnt to the responsive cells, which have traditionally been studied *in vivo*. Finally, we focus on advances in presenting Wnt ligands to the stem cells *in vitro* to study their function. In particular, we describe a system that can recapitulate aspects of the stem cell niche by providing localized Wnt proteins on synthetic surfaces. Localized Wnt proteins can affect cell fate decisions and control asymmetric cell division (ACD), processes essential for tissue formation and regenerative medicine applications.

## 2. Investigating Wnt signalling in the mammalian stem cell niche

Several methods have been implemented to identify Wnt-responsive stem cells in numerous tissues. Each experimental approach has its limitations, and therefore a combination of methods can improve the characterization of the stem cell compartment. For example, traditional *in vivo* functional assays such as the knockout and overexpression of Wnts or Wnt regulatory proteins (for example, the Wnt antagonist DKK) have been successfully used [9,40]. However, these may have off-target effects including a systemic influence on the physiology of the body. Additionally, the knockout of a Wnt gene in a subpopulation of cells may not yield an obvious phenotype [40]. This is often attributed to other Wnts expressed in the tissue that can compensate for the knocked out gene. Multimerized TCF sites or Axin2 based reporters that are fused to EGFP or LacZ can report on the activity of Wnt/β-catenin signalling [41–45] in identified stem cells. However, in the absence of stem cell markers and functional assays, employing these reporters to provide a proof of the stem cell identity can be challenging. Recent methods to label Wnt ligands and advances in microscopy have provided new insights into visualizing Wnts at the cellular level. These technologies coupled with other methodologies including *in situ* RNA hybridization and lineage tracing have advanced our knowledge of Wnt signalling in the stem cell compartment. As such, Wnt-producing cells and Wnt-responsive stem cells can now be detected in a variety of tissues of the body at a high cellular resolution.

### 2.1. Contemporary methodologies for investigating Wnt presentation and response

The best approach to study the localization of Wnt is by detecting the ligand directly. However, immunofluorescence methods have proved to be challenging. The majority of existing Wnt antibodies do not faithfully detect the protein *in situ*. Furthermore, visualizing Wnt proteins by fusing endogenous Wnt to a fluorescent protein tag is reported to produce Wnt proteins with lower activity [46], possibly due to disruption of disulfide bridges. Farin *et al.* [12] recently overcame this by genetically tagging Wnt3. A haemagglutinin (HA)-tag was introduced to a weakly conserved region in the N-terminus of the *Wnt3* locus, thereby generating a tagged full length *Wnt3* allele. The HA-Wnt3 protein expressed by knock-in mice did not display a deficiency in signalling activity. MacDonald *et al.* [47] have also successfully tagged V5 to the C-terminus of Wnts without an observable loss of activity, thereby providing a valuable tool to monitor Wnt dispersal in the stem cell niche.

Advances in the development of fluorescent probes could also be used for tagging Wnt proteins without compromising their activity. For instance, specific amino acids within a protein of interest can be genetically replaced in a site-specific manner by synthetic counterparts [48]. When incubated with the appropriate fluorescent conjugate, these synthetic amino acids bind to the probes and allow for precise detection of the protein of interest in living cells.

Utilizing these Wnt-tagging strategies in conjunction with advances in microscopy and tissue handling can yield a comprehensive view of the mode of Wnt presentation and dynamics within the tissue. For example, the development of lattice light-sheet microscopy (LLSM) can generate a three-dimensional (3D) image at a high spatio-temporal resolution [49]. LLSM uses an ultra-thin structured light sheet to rapidly slice through a specimen, exciting only the fluorescent probes in that specific plane. This is ideal for capturing fast, highly dynamic mechanisms *in vivo* with minimal photo-toxicity. Furthermore, the CLARITY technique facilitates imaging by replacing lipids with hydrogel-based structures. This modification renders the tissues transparent while retaining structural elements, proteins and nucleic acids [50].

Scientists have also used transcriptomics to identify potential Wnt-producing cells. Cellular transcriptomic profiling is a powerful tool to study Wnt expression. However, it is hard to identify the precise location of Wnt-producing and receiving cells once they have been extracted from the tissue. RNA *in situ* hybridization can circumvent this. In particular, recent technologies [51] that offer a high cellular resolution with the possibility to quantify transcripts have been used to detect Wnt transcripts in numerous niches (for example, the interfollicular and testicular niches) [9,24]. Importantly, Wnt transcripts (potentially in the Wnt-producing cell) can be co-detected with a Wnt target gene, such as *Axin2*, in the receiving cell to provide a picture on the Wnt-responsive stem cell compartment. However, RNA *in situ* does not report on the transcriptional regulation status of the transcripts (e.g. epitranscriptomics).

RNA *in situ* hybridization has often been used in conjunction with a lineage tracing approach to validate the identity of the stem cell compartment. Lineage tracing uses a Wnt target gene to irreversibly tag Wnt-responsive stem cells and their cell progenies *in vivo*. Frequently employed reporters include *Lgr5*, a G-protein coupled-receptor and Wnt enhancer, [52] or *Axin2*, a negative regulator of the Wnt signalling pathway [42]. For example, the Axin2 reporter is made by knocking in a tamoxifen-inducible Cre recombinase (*Cre^ERT2^*) to the endogenous target locus of embryonic stem cells (ESCs). Mice derived from the knock-in *Axin2^CreERT2^* ESCs are crossed with a reporter strain such as the fluorescently tagged Rosa26-mT/mG (R26R^mTmG^). Upon tamoxifen induction, Wnt-responsive cells [53,54] are fluorescently and irreversibly marked. Reporters used in the current literature for lineage tracing in various mammalian stem cell niches are summarized in table 1 [9,12,15,16,18,26,55]. The use of these techniques for investigating Wnt signalling in the stem cell niche is discussed in specific examples below.

### 2.2. Intestinal stem cell niche

The intestinal epithelium is composed of highly proliferative crypts and villi that protrude into the lumen. Over the course of 3–5 days, stem cells at the base of the colon crypt divide and give rise to differentiated cells that repopulate the villi. Wnt signalling is a key regulator of this process. Wnt target genes are expressed in a gradient where expression is highest in the crypt and is inversely correlated to cellular differentiation [13]. Disruption of Wnt signalling halts crypt proliferation, subsequently leading to loss of intestinal tissue and morbidity [56–58]. Recent studies that employ lineage tracing, organoid cultures and *in vivo* Wnt labelling have characterized a discrete Wnt-responsive stem cell population within the intestinal niche [12,13].

**Figure 1.**
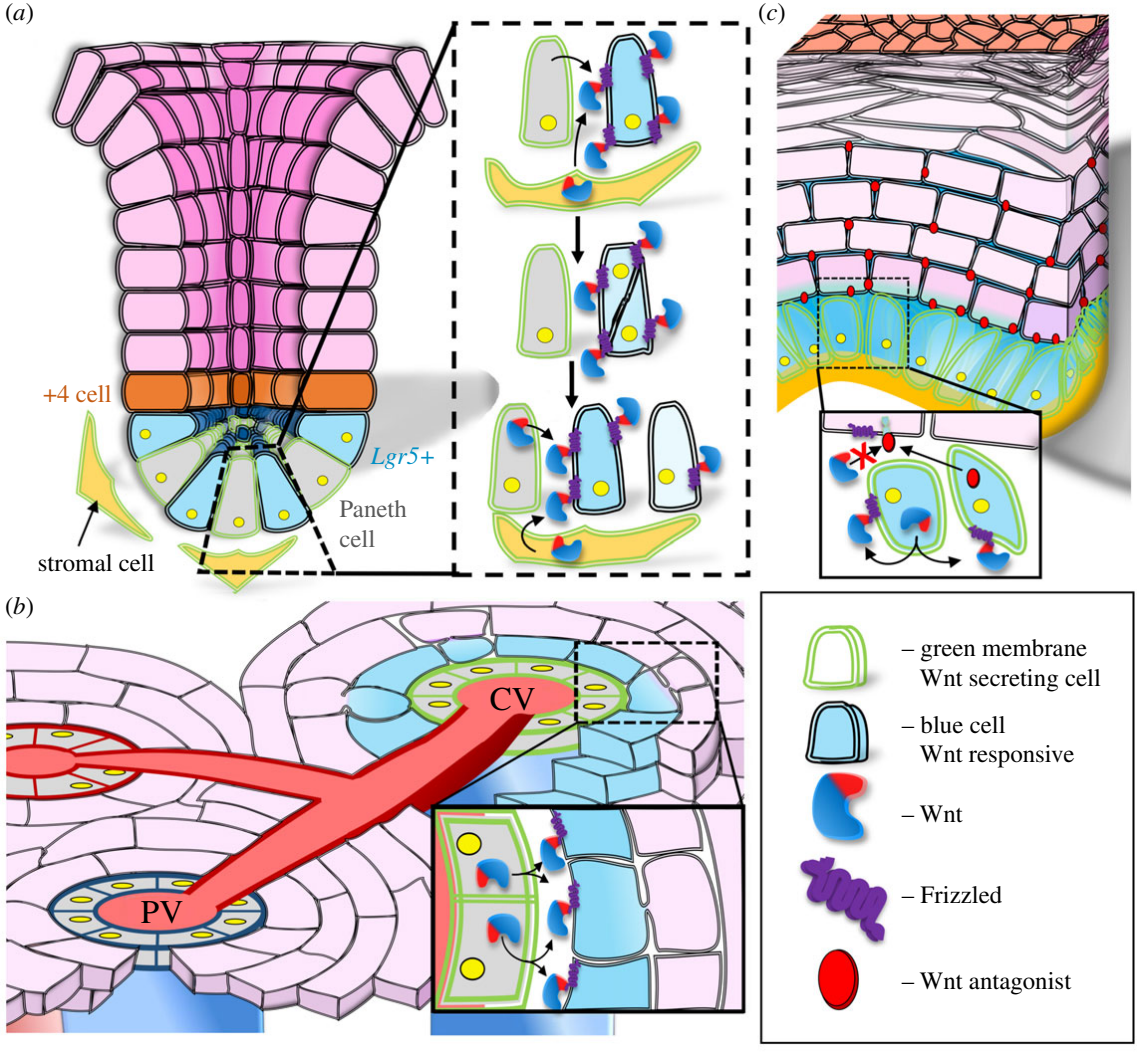
Wnt presentation within the mammalian stem cell niche. (*a*) The intestinal stem cell niche. Wnt is secreted from Paneth and stromal cells to a defined subset of Wnt-responsive cells (*Lgr5+*). *Lgr5*+ stem cells require direct contact with Paneth cells to receive Wnt signals, which are tethered to the stem cells by the Frizzled receptor (see inset). Following tethering, Wnt is distributed by stem cell division, producing a diluted Wnt signal. As cells divide and move out of the proliferative zone, away from a Wnt signal, they differentiate. (*b*) The pericentral hepatic progenitor niche. Paracrine Wnt signals are secreted from endothelial cells surrounding the central vein (CV) to adjacent hepatic progenitor cells (see inset). The descendants of the progenitor cells differentiate as they move away from the CV (and Wnt signal), towards the portal vein (PV). Modelled according to Wang *et al.* [16]. (*c*) The interfollicular stem cell niche. IFE stem cells can receive Wnt signals in an autocrine manner, while simultaneously secreting Wnt antagonists apically (see inset). These antagonists (e.g. DKK) diffuse into the suprabasal layers, to restrict Wnt signalling and promote differentiation.

Genetic labelling of *Lgr5+* cells identified the presence of highly cycling, Wnt-responsive stem cells. *Lgr5+* cells are spatially restricted to the base of the crypt with the ability to produce all intestinal cell types [13]. These stem cells are interspersed with Paneth cells (figure 1*a*), specialized descendants that act as a niche by providing Wnt signals to the stem cells [59]. Until recently, knowledge of how Wnt ligands are dispersed in the intestinal niche was limited. In organoid cultures derived from mice, using an HA-tagged Wnt3, Farin *et al.* [12] detected an enrichment of Wnt3 on the basolateral surface of *Lgr5+* cells. The authors propose that Paneth cells produce Wnt3 and transfer it to the adjacent *Lgr5+* stem cells. The Wnt–Frizzled complex on the membrane of *Lgr5*+ cells then disperses via cell division. This process dilutes surface-bound Wnt, therefore generating a Wnt gradient (figure 1*a*, inset). Highlighting this notion, inhibition of cell division blocked crypt formation and caused the retention of Wnt3 at Paneth cell membranes. The necessity of direct Paneth–*Lgr5*+ cell–cell contact is underscored by observations that *Lgr5*+ cells maximize their contact with Paneth cells [60].

Farin *et al.* [12] also show that the Wnt3 signal typically penetrates up to two cells in diameter, indicating a tightly controlled, spatially restricted signalling niche. The importance of this is highlighted by deletion of adenomatous polyposis coli (APC) in mice, which deregulates Wnt signalling by activating the pathway in the absence of a Wnt ligand. APC deletion leads to *Lgr5+* cell specific adenoma formation in mice [55]. Owing to their high rate of cell division and proximity to Wnt signals, *Lgr5+* cells possess a higher tumorigenic potential than differentiated cells. Indeed, during tumorigenesis *Lgr5+* cells can act as cancer stem cells in intestinal adenomas, giving rise to progeny cells with active Wnt/β-catenin signalling [55,61].

Intriguingly, it has also been reported that macrophages and subepithelial mesenchymal cells secrete Wnts that support the intestinal niche, particularly in response to injury [62–64]. How cells of the stroma present these secreted Wnts is currently unknown. Interestingly, in the ‘+4’ position, above the *Lgr5+* stem cell zone, resides a functionally distinct population of slowly cycling stem cells. These cells are not responsive to Wnt. The cells in the ‘+4’ position have been described through labelling with numerous markers (*Lrig1*, *Bmi1* and *mTert*) [65–67] and are proposed to be responsive to the ErbB signalling pathway. *Lgr5+* stem cells and cells in the ‘+4’ position are multipotent, with the ability to generate all the epithelial lineages of the intestine [67,68]. The relationship between these two stem cell populations is not fully understood. However, it is suggested that *Lgr5+* stem cells are mainly required for intestinal maintenance during homeostasis, while +4 stem cells are activated in response to tissue injury. Moreover, when the crypt is damaged the +4 stem cells can convert to *Lgr5+* stem cells [69,70].

Despite these recent breakthroughs, the mechanisms and impacts of Wnt presentation in the intestinal niche have not been fully elucidated. For example, *Lgr5+* cells residing next to +4 stem cells at the edge of the niche contact a single Paneth cell (figure 1*a*), thereby receiving a localized and directional Wnt signal. *In vitro*, an orientated Wnt signal induces ACD [4]. The implication of the oriented Wnt signal on the intestinal niche is yet to be determined.

### 2.3. Pericentral hepatic progenitor niche

The liver has long been known to possess considerable regenerative potential. Until recently, in the uninjured state, this property was attributed to the proliferation of existing hepatocytes. This capacity to regenerate is possible despite the limited replicative capability of hepatocytes due to their polyploid nature, and heterogeneity in both age and function [71–73]. Recent lineage tracing studies, however, have identified a population of diploid, Wnt-responsive cells, capable of repopulating the liver with all hepatocyte lineages under homeostatic conditions (figure 1*b*) [16]. This study exploited the genetic labelling of a population of *Axin2+* cells, residing adjacent to the central vein. Descendants were traced *in vivo* from the central vein, towards the portal vein over the period of a year, and sometimes were observed to comprise entire lobules. *Axin2+* cells also persisted around the central vein over this time period, indicative of their self-renewal.

Wang *et al.* [16] used *in situ* RNA hybridization to identify the expression of Wnt2 and Wnt9b in endothelial cells localized exclusively around the central vein and in close proximity to *Axin2+* cells. These cells are likely to contribute to the progenitor niche. Furthermore, Wnt secretion has been demonstrated to be essential for the maintenance of *Axin2+* cell function. For example, inducible Wntless (WLS, a protein crucial for the transport of Wnt to the plasma membrane, discussed in more detail below) knockout mice displayed a sharp reduction in Axin2 expression, coinciding with decreased proliferation and loss of pericentral hepatocyte function. This suggests that short-range Wnt paracrine signals disperse from central vein endothelial cells to adjacent stem cells (figure 1*b*, inset). Supporting this notion, *Axin2+* descendants located farther from the central vein and Wnt source reportedly become polyploid and express differentiation markers [16].

Several studies have also implicated Wnt/β-catenin signalling in liver maintenance, metabolic zonation and regeneration [74–86]. Planas-Paz *et al.* [17] suggested that Wnt receptors and ligands are broadly expressed in liver compartments. The authors have also identified *Lgr4*+ hepatocytes throughout the liver as cells that contribute to liver maintenance during homeostasis and regeneration. Unlike the aforementioned pericentral *Axin2*+ cell linage tracing experiments, pericentral *Lgr5*+ hepatocytes studied in Planas-Paz *et al.* [17], which also express Axin2 transcripts, do not appear to proliferate or give rise to hepatocytes during homeostasis or regeneration. The differences between the findings have yet to be reconciled.

### 2.4. Interfollicular epidermal stem cell niche

Cells from the surface of the interfollicular epidermis (IFE) are constantly shed and repopulated by stem cells in its basal layer. Lineage tracing studies in mice, using the *Axin2* reporter, demonstrated that these stem cells are Wnt responsive and can generate labelled clones throughout the epidermis for up to a year (figure 1*c*) [9]. This study is supported by findings that β-catenin is required for maintenance of IFE stem cells both *in vitro* and *in vivo*. For example, utilizing a dominant negative form or deletion of β-catenin leads to a decrease in stem cell proliferation, coupled with an increase in differentiation [87,88]. Additionally, deletion of the Wnt signalling transcription factors TCF3/4 or induction of Wnt inhibitor DKK *in vivo* stunts IFE proliferation and results in reduction of skin thickness [88,89].

The stem cells in the IFE niche are also Wnt-producing cells (figure 1*c*, inset). RNA *in situ* hybridization experiments have shown that the same stem cell expresses both Axin2 and Wnt4 or Wnt10 [9]. Concurrently, IFE stem cells also produce Wnt antagonists that are reported to diffuse apically, dampening the Wnt signals in the suprabasal layers. Indeed, RNA *in situ* hybridization in the IFE niche detects Axin2 and DKK3 production in the basal layer. Immunofluorescence shows that DKK3 is localized to differentiating keratinocytes outside the stem-cell compartment [9]. This suggests that the IFE niche capitalizes on the signalling ranges of its regulators to maintain appropriate levels of self-renewal and differentiation. Short-range Wnt signals are highly concentrated at the basal layer, while diffusion of Wnt antagonists spatially restricts differentiation to the suprabasal layers (figure 1*c*).

Taken together, research to date shows that Wnts are critical for maintaining homeostasis within the stem cell niche. Despite this, research on Wnt signal presentation and reception in stem cell niches is still in its infancy with many questions remaining unanswered. Next, we will review known mechanisms by which Wnt ligands are presented and received by responsive cells, as well as advances in technologies that can help us to further elucidate Wnt pathways in the niche.

## 3. Processing and presentation of Wnt ligands

**Figure 2.**
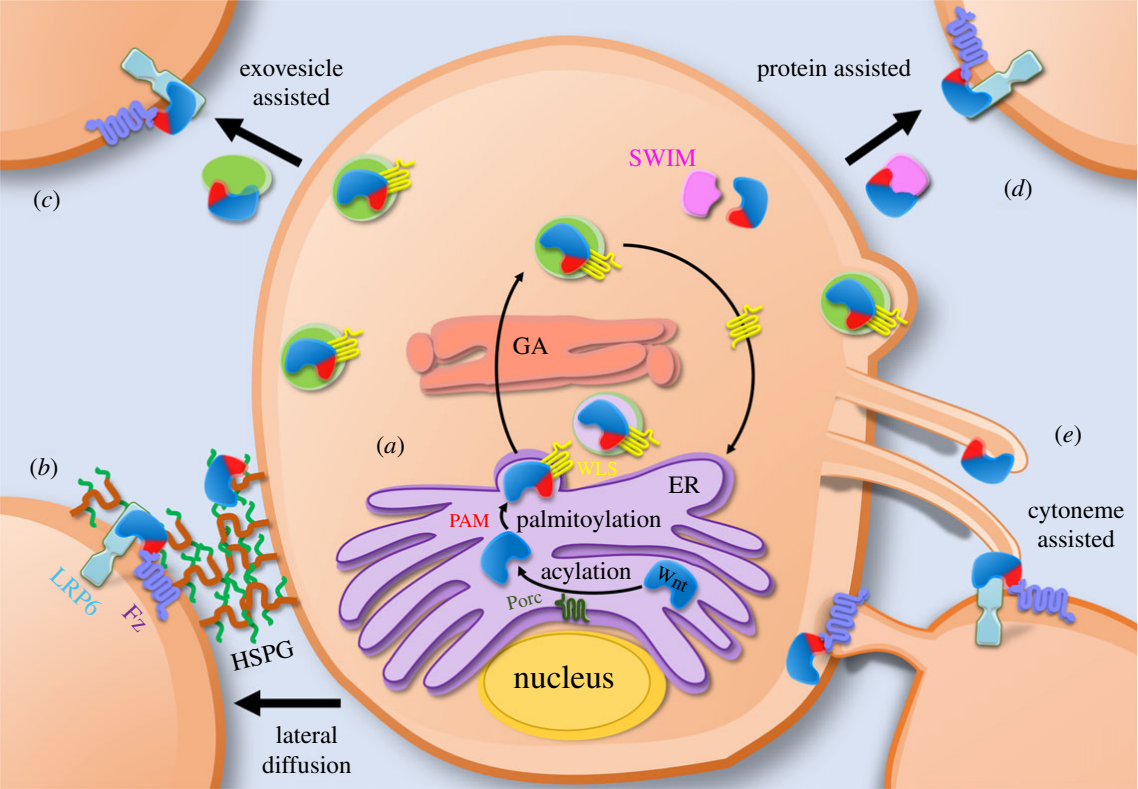
Schematic of Wnt processing and intra- and extracellular transport. (*a*) Wnts are acetylated by Porcupine (Porc) in the ER, catalysing the addition of the lipid moiety palmitoleate (PAM). PAM facilitates an interaction between Wnt and WLS, which is required for the transport of Wnt proteins (Wnts) through the secretory pathway to the plasma membrane. (*b*) Lateral diffusion. For example, heparin sulfate proteoglycans (HSPGs) enable restricted diffusion of Wnts to adjacent cells. (*c*) Exovesicle assisted. Wnts are distributed to recipient cells via figure 2 exosomes. Wnts bind to the surface of exosomes with their palmitoleate most likely inserted in the lipid bilayer. (*d*) Protein assisted. Wnts bind to specific transport proteins (e.g. SWIM), which can shield palmitoleate enabling Wnt solubility and transport to receiving cells. (*e*) Cytoneme assisted. Wnts are mobilized to the tips of actin-based filopodia, which extend through the extracellular space to activate signal transduction in recipient cells. Wnt-receiving cells can also extend Frizzled decorated cytonemes to Wnt-producing cells. GA, Golgi apparatus.

Precise targeting of Wnt proteins to receiving cells within the stem cell niche is vital to retain the balance between self-renewal and differentiation. To attain correct targeting, Wnt-producing cells use several mechanisms to present the secreted protein. Prior to secretion, the majority of studied Wnt ligands undergo post-translational modification [46,90,91]. Wnts are acetylated at a conserved serine residue in the ER by the membrane associated *O*-acyl transferase, Porcupine [92] (figure 2*a*). This modification is crucial for the addition of the lipid moiety palmitoleate, which is essential for Wnt secretion [93] and Frizzled receptor binding [32]. Mutation of serine 209 (required for acylation) in human Wnt3a significantly restricts its secretion and dampens the signalling activity of the remaining secreted protein [32,93,94]. Most Wnts are also glycosylated, but the functional relevance of this remains contentious [31,95,96].

Newly synthesized Wnts have a targeting sequence that directs them to the ER [97], where the addition of palmitoleate facilitates an evolutionarily conserved interaction between Wnt and WLS [92]. The Wnt–WLS interaction is required to transport Wnt through the secretory pathway to the plasma membrane. The importance of WLS is reflected in studies showing that mutation of a conserved Wnt serine residue (serine 209 in human Wnt3a) that is essential for Wnt–WLS interaction, or the deletion of WLS results in cellular retention of Wnt [98–100]. The mechanism by which WLS chaperones Wnt from the secretory pathway to the plasma membrane and primes it for secretion is only now becoming clear (reviewed in [90]). This is possible because the mechanism is Wnt ligand specific and unique to the cellular architecture in each stem-cell niche.

The addition of palmitoleate confers hydrophobicity to Wnts, imposing constraints on solubility and signalling range. This is ideal for spatially restricting the signal to a limited number of cells that are in the proximity of the niche. Wnt signals can undergo lateral diffusion, where Wnt is transferred to adjacent cells. This diffusion does not occur freely throughout the extracellular matrix and instead is spatially confined. In some cases, cell surface proteins like heparin sulfate proteoglycans (HSPG) mediate the transfer of Wnt ligands within the extracellular matrix (figure 2*b*) [101,102]. Studies utilizing mutant *Drosophila* HSPGs, Dally and Dally-like, suggested that HSPGs form a scaffold to support the transfer of Wnts between cells [101]. Differential HSPGs expression may offer an additional mechanism to modulate Wnt transfer. Moreover, *in vitro* studies have shown the importance of HSPGs in maintaining the solubility and activity of purified Wnt proteins [103].

Wnts have also been observed outside the predicted range of diffusion, implying that other mechanisms must be employed to circumnavigate their hydrophobicity for long-range transport. Biologically active human Wnt3a and *Drosophila* Wnt (known as Wingless, Wg) have been detected in exosomal fractions from the supernatant of cultured cells [104,105]. This provides a viable mechanism for long-range Wnt transport. Wnts may associate with the extracellular surface of exosomes with their palmitoleate group shielded by the lipid bilayer (figure 2*c*). Studies also suggest that Evi, the WLS *Drosophila* homologue, is required to shuttle Wg into multivesicular bodies (MVBs) within the wing disc. MVBs then fuse with the plasma membrane for secretion as exosomes [105]. Furthermore, exosomal Wnt transport has been implicated in the neuromuscular junction [106,107].

Transport proteins that form complexes with Wnt, and presumably shield the lipid moiety, have been identified. For example, secreted Wingless-interacting molecule (SWIM), from the lipocalin protein family, has been shown to bind directly to *Drosophila* Wg in a lipid-dependent manner. This interaction facilitates Wg solubility, enabling long-range transport while still maintaining Wg activity (figure 2*d*) [108]. Deregulation of SWIM by RNAi results in loss of Wg long-range signalling in wing discs, whereas short-range signalling and secretion remain intact. Recently, Alexandre *et al.* [28] demonstrated that restriction of Wg to the cell membrane had no impact on wing patterning. This suggests that long-range Wg transport is not in fact required for *Drosophila* wing development. Instead, the authors proposed that initial ubiquitous expression of Wg throughout the wing is adequate for further development. Soluble Frizzled related proteins (sFRPs) are also reported to increase the signalling range of Wnts [109]. This initially seems paradoxical, as sFRPs are typically seen as Wnt inhibitors. However, Mii & Taira [109] propose that HSPGs compete with sFRPs for Wnt binding. Therefore, short-range HSPG signalling might be blocked while sFRP-bound Wnt diffuses through the extracellular matrix.

Another mechanism for spatially restricted distribution of Wnt ligands involves cytonemes (reviewed in detail in [110]). Cytonemes are thin, tubular actin-based filopodia with the ability to mobilize signalling molecules for contact-dependent signal transduction. Initial observations identified the localization of *Xenopus* Wnt2b and zebrafish Wnt8a clusters to cellular extensions [111,112]. Wnt8a clusters in particular can activate the filopodia nucleation complex through recruitment of the transducer of Cdc42-dependent actin assembly protein 1 (Toca-1) [113]. These cytoneme-like filopodia were observed to elongate with concentrated Wnt8a clusters at the tip (figure 2*e*) and contact the soma of Wnt-responding cells via LRP6 receptors for signal transduction.

Cytoneme-like filopodia are implicated in the intracellular transport of Wnt ligands for embryonic anteroposterior patterning in zebrafish embryos. In this system, extending and shortening filopodia length deregulates Wnt distribution, leading to opposing effects on neural plate patterning [113]. Interestingly, Barker *et al.* [13] observed apical extensions of cytoplasm in *Lgr5+* cells in mouse intestinal crypts. Independent of this observation, Lgr5 has been reported to drive the formation of cytoneme-like structures with the ability to deliver signalling effectors [114]. This potentially indicates a cytoneme-mediated mechanism of Wnt signalling in the intestinal stem cell niche. Wnt signalling components have also been detected on cytonemes from Wnt-receiving cells. *Drosophila* flight muscle progenitors send out Frizzled decorated cytonemes towards Wnt secreting cells in the wing imaginal disc to capture Wnt ligands. These Wnt/Frizzled complexes are then transported to the soma in a retrograde direction [115].

Overall, the operation of Wnt transmission might be tissue, cell type and Wnt specific. It may also be differentially employed during homeostasis, injury and disease. Identification of Wnt-responsive stem cells in combination with *in vivo* knowledge of Wnt presentation and Wnt protein purification have enabled the evolution of technologies for tissue engineering. Purified Wnt ligands can now be used as platforms to culture, expand and direct the differentiation of stem cells into tissue-like structures.

## 4. *In vivo* presentation of Wnt proteins: advancements towards tissue engineering

To study the direct effect of Wnts on stem cells, *in vitro* assays provide a valuable platform. While powerful genetic tools can be used to manipulate Wnt signalling, studies of hydrophobic Wnt proteins have been hampered by the technical challenges of purification and by their localized action. Traditionally, studies have primarily focused on methods that activate the Wnt pathway in an untargeted manner. This includes the addition of small regulatory molecules, which activate Wnt signalling downstream of receptor binding, such as CHIR990021 and BIO, GSK-3β inhibitors [116–119] and IWR, a stabilizer of Axin and the destruction complex [120]. Nanoparticles coupled to antibodies targeting the Frizzled2 receptor have also been used to stimulate Wnt signalling through a subset of receptor complexes [121]. These reagents have proved to be useful but can only activate parts of the pathway and can have off-target effects including the activation of other pathways. More recently, advances in Wnt ligand purification and delivery have enabled spatial control of the Wnt signal, more faithfully replicating what occurs *in vivo*. These advances provide insight into Wnt activation at the single cell level and can be used for tissue engineering and regenerative medicine applications.

### 4.1. Non-directional presentation of Wnt ligands

**Figure 3.**
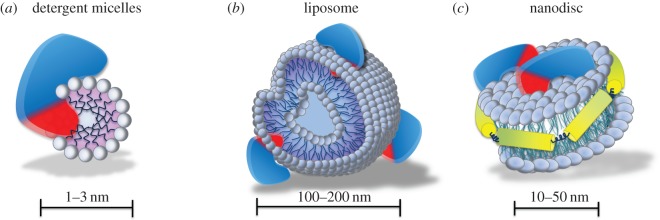
Schematic representation of non-directional delivery of Wnt. (*a–c*) Wnt can be solubilized by detergent micelles (*a*), or carried on liposomes (*b*) or nanodiscs (*c*). Hydrophobic moiety of Wnt (e.g. palmitoleate) is represented in the schematic by red.

Wnt-conditioned media produced by Wnt secreting cells have been used to activate Wnt signalling in cell culture. However, Wnt ligands are presented to the responsive cells in a non-directional manner. Moreover, precautions must be taken when interpreting these findings as Wnt-conditioned media contain other secreted molecules, which may affect cellular responses. Additionally, this method does not allow for precise control over the Wnt protein concentration, important for the cellular response [122] and expanding stem cells *in vitro* [3]. Purification of Wnt proteins [123–125] has been a breakthrough in the field. Through the use of these purified ligands, embryonic and adult Wnt-responsive stem cells, including mouse ESCs, mammary gland, neuronal and intestinal stem cells have been isolated and expanded [3,6,126,127]. This method has paved the way for investigating general mechanisms of cellular maintenance and differentiation. Importantly, purified recombinant Wnts must be stored in detergent to maintain activity and solubility (figure 3*a*). However, this can be toxic to some cells.

Lipid-based systems such as liposomes have been used to solubilize Wnt for *in vitro* and potentially *in vivo* delivery without the use of detergents [128,129]. Liposomes are typically spherical vesicles comprising an aqueous core enclosed by a lipid bilayer. Wnt proteins have been packaged into liposomes in a manner that tethers the protein to the liposome surface. Wnt ligands retain their proper protein folding and biological activity, possibly by shielding the lipid moiety within the bilayer of the liposomes (figure 3*b*). More recently, murine Wnt3a has been integrated into nanodiscs, which comprise a phospholipid bilayer and an apolipoprotein A-I scaffolding component. As in liposomes, Wnt is proposed to bind the lipid surface of the nanodisc, with the palmitoleate group inserted into the lipid bilayer. This solubilizes Wnt and simultaneously maintains its activity (figure 3*c*). Wnt3a nanodiscs have been used for *ex vivo* delivery into hematopoietic stem and progenitor cells (HSPCs) [130]. These Wnt3a nanodiscs can activate the Wnt/β-catenin pathway and stimulate the proliferation and expansion of HSPCs [130]. Nanodisc size is advantageous compared to liposomes. Liposomes generally have a diameter within the range of 100–200 nm, while nanodiscs tend to be much smaller at 10–50 nm in diameter, thereby allowing for more efficient penetration of the cellular environment [131]. Lipid-based delivery of Wnt does, however, have a major drawback as introducing lipid biomolecules may affect cellular responses. For example, although an empty nanodisc vector has no effect on stimulating Wnt signalling, it does elicit HSPC cell proliferation and expansion [130].

The recent crystal structure of *Xenopus* Wnt8 [32], in complex with the Frizzled 8 cysteine-rich domain, revealed that the palmitoleic acid lipid group moiety on Wnt directly interacts with the receptor. This interaction is essential for transducing the signal. The mechanism by which the Wnt on the liposomes or nanodiscs exposes the palmitoleate to interact with Frizzled remains elusive.

The aforementioned methods present Wnt in a non-directed manner to stem cells. Evolutionarily, Wnt is seen as a symmetry-breaking cue [1,27] and *in vivo* Wnt proteins are often secreted locally and presented in a spatially restricted manner to the responsive cell [1,12,28–30]. *In vitro* methods that control Wnt signal presentation in a localized manner can better mimic cellular niches [132] and provide possibilities to investigate how cells interpret this positional cue. In the next section, we discuss methods to achieve this through the immobilization of an active Wnt signal to synthetic surfaces. These immobilization techniques provide a sustained, covalently bound and active Wnt signal.

### 4.2. Constructing a localized Wnt signal niche on synthetic surfaces

**Figure 4.**
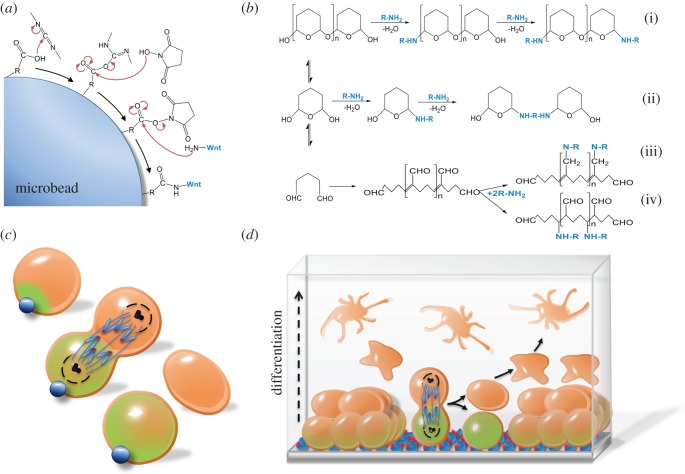
Wnt immobilization on synthetic surfaces for targeted delivery. (*a*) Covalent binding of Wnt to a microbead. Representation of the chemistry for covalent linkage using activated carboxylic acid. (*b*) Covalent binding of Wnt to a basal surface. Representation of possible reactions used to covalently bind Wnt to a basal surface using glutaraldehyde. Under neutral pH conditions, oligomeric hemiacetal or monomeric hemiacetal forms of the glutaradehyde can react with the functional group on the surface and/or protein (i–ii). Other reactions include Schiff-base reaction (iii) and Michael-type reaction (iv). ‘R’ represents Wnt protein or the backbone of the functional group on the surface. (*c*) Representation of Wnt-microbead inducing oriented ACD. Wnt-microbead polarizes the Wnt/β-catenin signalling machinery (green shading) in a single mouse embryonic stem cell. Polarization proximal to the Wnt-microbead is retained throughout cell division, giving rise to two daughter cells. The Wnt-proximal cell retains a high level of pluripotency markers, while the Wnt-distal cell adapts an epiblast stem cell fate. Concurrently, inheritance of the mother centriole (indicated by appendages) is observed in the Wnt-proximal cell. (*d*) Representation of a Wnt basal surface adapted to a 3D system to recapitulate a bone stem cell niche *in vitro*. hMSCs are seeded on a Wnt immobilized basal surface overlaid with a 3D collagen layer. Localized Wnt maintains the hMSC population while directing cellular migration and increasingly differentiating osteognic cells into the gel.

We have shown successful immobilization of Wnts on synthetic surfaces to mimic localized Wnt presentation within the stem cell niche. Covalent binding in a manner that does not disrupt the tertiary structures, in particular the disulfide bridges, is essential for maintaining signalling activity. We have previously described two approaches to immobilize Wnt ligands. Micro-beads coated with carboxylic acid can be converted to a succinimide ester in acidic pH [4] to facilitate covalent binding of Wnt to the bead (figure 4*a*). Glutaraldehyde-coated surfaces can also be utilized to immobilize Wnt through a reaction of the nucleophilic groups on Wnt proteins such as amine, thiol, phenol and imidazole [132–134] (figure 4*b*). Glutaraldehyde exists in aqueous solutions in a mixture of monomeric and polymeric states and the relative proportion of these states is pH-dependent [135]. This feature allows for multiple reactions with the Wnt protein including aldol condensation, Michael-type addition and Schiff-base reactions (figure 4*b*). The Schiff-base reaction is unstable and therefore unlikely as the Wnt bound to the synthetic surfaces is highly stable and can be stored long term. Importantly, covalent immobilization of the Wnt eliminates the need for detergent to maintain the biological activity of the protein.

ESCs primarily divide symmetrically and can be expanded when purified Wnt proteins are added globally to culture media [3]. To mimic an *in vivo* situation where Wnt is presented to one side of the stem cell [1,12,28–30], we introduced the Wnt3a-microbead to a single mouse ESC. Contact with a Wnt3a bead prior to ESC division polarizes elements of the Wnt/β-catenin pathway including receptors LRP6 and Frizzled1, β-catenin and APC towards the bead. This distribution is maintained during and after cell division, giving rise to two daughter cells with different protein expression profiles (figure 4*c*) [4]. Subsequent analysis of pluripotent gene expression revealed that upon cell division, the cell proximal to the Wnt-bead retains markers of pluripotency. In the distal cell, these markers are downregulated and markers of epiblast stem cells are upregulated. These findings support a role for orientated presentation of Wnt in cell fate determination and ACD [4].

Time-lapse imaging also showed that localized Wnt ligands dictate the location and the inheritance of the mother centrosome (figure 4*c*) and orient the plane of mitotic division. For the first time, unlike non-directional Wnt, localized Wnt has been shown to affect mammalian stem cells by orienting ACD. How Wnt signalling induces ACD, a process essential for tissue development and regeneration, is yet to be fully elucidated; however, some clues do exist. β-catenin polarization correlated with an asymmetric inheritance of the mother centrosome [4]. This is an intriguing finding given that both β-catenin and APC comprise the mother centrosome, and interact with components of the mitotic spindle [136,137]. Whether the processes of centrosome and other organelle inheritance, spindle orientation and cell fate are coupled remain to be investigated.

We have recently expanded the immobilization technology through the use of glutaraldehyde chemistry to develop a novel and highly stable Wnt-platform. Wnt proteins have been immobilized to a basal surface which can induce, enrich and expand a monolayer of Wnt-responsive stem cells in culture [134]. The surface can be stored for months, and in culture induces Wnt signalling over multiple days. This means tissues can be isolated and enriched for Wnt-responsive stem cells in a short period of time without the need for genetic manipulation, cell sorting or advanced equipment. This is an important advancement in the field.

By combining the new Wnt-platform with basic knowledge of Wnt-mediated asymmetric stem cell division it is possible to efficiently engineer tissues *in vitro*. For example, we successfully adapted this platform into a 3D system to recapitulate a bone stem cell niche. Wnt proteins are known to play an important role in the biology of mesenchymal stem cells and bone development [138]. On the Wnt surface, we overlaid cultured bone marrow derived human mesenchymal stem cells (hMSCs) with a type 1 collagen gel, the main protein found in bone. Wnt ligands immobilized to the basal surface provided a spatially orientated signal to the cells. Over the course of several days, these cells were able to self-renew and generate organized and increasingly differentiating multicellular osteogenic cell layers (figure 4*d*). Formation of mineralized nodules on the upper layers of the Wnt-platform gel was also observed. These findings are unique and illustrate a potential for controlling tissue orientation and organization using a localized Wnt cue [134].

By employing immobilized Wnt proteins, isolated Wnt-responsive stem cells and an appropriate 3D scaffold, we can potentially engineer organized 3D tissues that maintain the stem cell population. Tissue engineering can be used to study the biology of the human stem cell niche to determine the cellular cues required for tissues to form in 3D. Furthermore, these tissue structures can be used to model diseases and understand the processes behind defective tissue formation. Findings from these studies can also be harnessed for later implementation in pharmacological studies and tissue transplantation.

## 5. Concluding remarks

The stem cell niche is vital for regulating the stem cell compartment during tissue homeostasis and regeneration. Key to this regulation is the spatial restriction of signalling molecules, which can be used to delineate cellular identity. Within the niche, where the signalling molecules are concentrated, a defined stem cell zone for self-renewal is maintained. Cell movement out of the signalling range facilitates cellular differentiation. In many tissues, the short-range dispersal of Wnts and their secretion from a localized, finite set of cells make Wnt ligands ideal signalling candidates to control the stem cell compartment. The Wnt signalling pathway provides essential cues for cell fate determination.

Studying the secretion mode of Wnt ligands, their spatial distribution and effect on the stem cell function and tissue architecture has provided insights into how to reconstruct the Wnt niche *in vitro*. By using purified Wnt ligands and protein immobilization techniques, fundamental questions in developmental biology, including the mechanisms behind asymmetric stem cell divisions, can be answered. This knowledge can be harnessed and applied to engineer 3D human tissues *in vitro*. Modelling human tissues will be beneficial for studying the basics of tissue formation and regenerative medicine purposes.
